# Multi-Modal Courtship in the Peacock Spider, *Maratus volans* (O.P.-Cambridge, 1874)

**DOI:** 10.1371/journal.pone.0025390

**Published:** 2011-09-27

**Authors:** Madeline B. Girard, Michael M. Kasumovic, Damian O. Elias

**Affiliations:** 1 Department of Environmental Science, Policy and Management, University of California, Berkeley, California, United States of America; 2 Evolution & Ecology Research Centre, University of New South Wales, Kensington, Sydney, Australia; Monash University, Australia

## Abstract

The peacock spider, *Maratus volans*, has one of the most elaborate courtship displays in arthropods. Using regular and high-speed video segments captured in the lab, we provide detailed descriptions of complete male courtship dances. As research on jumping spiders has demonstrated that males of some species produce vibrations concurrently with visual displays, we also used laser vibrometry to uncover such elements for this species. Our recordings reveal and describe for the first time, that *M. volans* males use vibratory signals in addition to complex body ornaments and motion displays. The peacock spider and other closely related species are outstanding study organisms for testing hypotheses about the evolution and functional significance of complex displays, thus, this descriptive study establishes a new model system for behavioral ecology, one that certainly stands to make important contributions to the field.

## Introduction

Research on animal courtship has demonstrated that males of many species produce elaborate multi-component signals spanning more than one sensory modality (multi-modal signals e.g. combinations of tactile, visual, acoustic, etc. signals). The adaptive significance of multi-modal signal structure, however, is not well understood. For instance, each component of multi-modal signals may be informative to females in a different way (multiple message hypothesis [1]). In contrast, different multi-modal signal components may independently reflect the same information, providing back-up for intrinsic signaling errors (redundant signal hypothesis [1]). Moreover, females may evaluate only one, or a few, traits at a time with complex male signals; or instead, they may process many signal components together to facilitate the evaluation of potential mates [1]–[3]. Although complex signaling has become a recent focus of much communication research, careful dissection of signaling behavior and the signals involved in mating interactions is often missing from these studies. Additionally, biases in human senses have led to an oversimplification in the potential information contained in animal signals [4]–[5], and in some instances even failed to identify the modalities and signals most involved in female choice [6]–[8]. Comprehensive study of the signals themselves is an overlooked, yet crucial component of animal behavior research.

Jumping spiders (Family: Salticidae) are visual specialists among the arthropods [9]. Not surprisingly, in the majority of species examined to date, males possess sex-specific visually-mediated displays that are important during courtship [10]–[15]. Substrate-borne vibrations, in conjunction with visual displays, have also been demonstrated to function in jumping spider courtship [15]–[23] and are important for mating success in a number of species [6], [23]–[25]. In particular, those in the genus *Habronattus* are well known to communicate using a dynamic repertoire of both visual displays and intricate vibrational signals [17], [26]–[27]. Intense sexual selection is predicted to lead to the evolution of such complex displays [28] and has also been implicated as being an important driver of diversification in jumping spiders [29]–[32].

Although male salticids are often highly ornamented relative to their female counterparts, the Australian endemic peacock spider (*Maratus volans*) stands out as an exceptional example. During courtship, a male peacock spider unfurls its brightly colored opisthosomal flaps, which are typically kept tucked around the abdomen [33]. The whole structure, which bears resemblance to the fan of a peacock, is then waved at a female in synchrony with an ornamented 3^rd^ pair of legs.

Despite the charismatic nature of the *Maratus* genus, virtually no work has been conducted on the displays of these species, including, *Maratus volans* [33]. However, based on the diversity of their behavior, particularly the species-specific mating displays that are likely to exist [34], research on *Maratus* promises to yield important insights on patterns of signaling and signaling complexity. Accordingly, we set out to uncover the complete repertoire of behaviors these males utilize during courtship. To characterize and quantify all male courtship displays and vibrational signals, we used regular and high-speed video as well as laser vibrometry. In this paper we describe, in detail, the remarkable courtship display of the peacock spider, *Maratus volans*. We show that males of this species make use of both visual and vibratory modalities in their courtship efforts. Distinct components of male behavior emphasize different aspects of a male's morphology and each display element consists of a unique combination of visual and vibratory signaling. These behavioral descriptions provide the necessary foundation for future work on *M. volans* as well as the entire *Maratus* genus.

## Methods

### Ethics Statement

All necessary permits were obtained for the described field studies: New South Wales National Parks and Wildlife Service license to MMK (# S12762).

### General Methods

Specimens were collected around the Sydney, New South Wales, area (field sites: Ku-ring-gai Chase National Park, and Cowan Field Station in the Muogamarra Reserve) during October and November of 2009. Live spiders were housed in individual containers and kept in the lab on a 12-hour on/off light cycle. Spiders were fed weekly a diet of fruit flies (*Drosophila melanogaster*) and occasionally crickets (*Acheta domesticus*).

First, live mature males (N = 11) and females (N = 10) were paired randomly between the hours of 09:00–15:00, and interactions between the pairs recorded on a digital VCR (Sony DVCAM DSR-20 digital VCR). Visual and vibratory courtship display elements of males were captured using a JAI CCD camera (CV-S3200) and a Polytec Scanning Laser Vibrometer (PSV-400, digitized at a 48.1 kHz sampling rate), respectively. Courtship recordings were conducted on an arena consisting of nylon fabric stretched over a circular wooden needlepoint frame (diameter: ∼27 cm). This fabric was used as it has been shown to pass frequencies with minimal distortion [27]. The arena was situated on wooden dowels (height: ∼7.5 cm) atop a larger rotating, circular platform (diameter: ∼35 cm). The camera was stationary, so rotation of the circular platform allowed us to keep males in the recording frame as they moved around the arena after females. Several square pieces of reflective tape (area: ∼1 mm^2^) were stuck to the surface of the nylon fabric, at the center of the arena, to serve as measurement points for laser vibrometry recordings. In between use, our arenas were cleaned with 75% ethanol to remove any chemical traces of previously run spiders.

One of the main merits of this set up was that males and females were allowed to move about freely on the arena and thus interactions would be more similar to those in the wild. In that sense, these recordings were helpful for making preliminary observations and capturing the overall progression of male visual displays and vibrational signals. However, because it is very difficult to maintain focus on moving spiders, this setup was not ideal for capturing entire courtship displays. Therefore, additional recordings were employed, similar to techniques used by Elias *et al.* [27].

**Figure 1:**
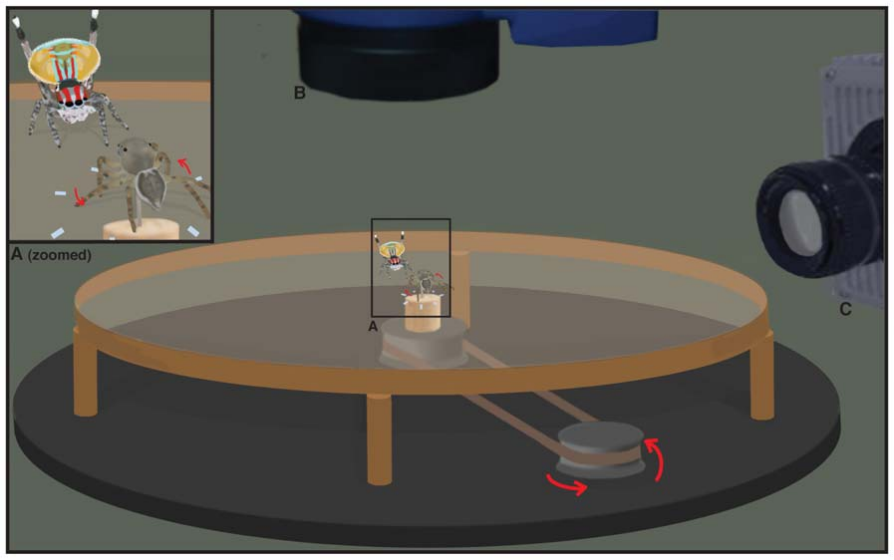
Courtship recording set-up. (A) Focal area for the regular speed camera (CV-S3200), (B) laser vibrometer (PSV-400) and (C) high-speed camera (SA3).

Instead of live females, dead female models were used to elicit males to court in a more contained and determined area. Conspecific female models were prepared by attaching freshly dead females to an insect pin, using melted bee's wax on the ventral surface of the cephalothorax. The previous arena set-up was modified so that the female models could be positioned at the center, atop the nylon fabric near the pieces of reflective tape. Glued to the top of the larger platform was a belt-pulley system with one pulley attached at the center of the platform (Fig. 1). On top of the centrally placed pulley we attached a small piece of cork to which female models could be attached and swiveled by rotating the other pulley, which was situated at the outermost part of the larger platform. This arrangement allowed us to move female models in a “lifelike” manner, which helped to entice males to court. One at a time, males (N = 11) were dropped into the arena and allowed to court freely. If males did not notice the female, mounted females were rotated until males took an interest and started to approach. Females were then positioned as if they were observing a male's activity head on. If males stopped courting for more than several minutes, female models were rotated slightly, in order to draw the male's attention. Pinned females were placed in the freezer at the end of each day for preservation, and were used for 1–2 weeks maximum.

Video and laser recordings were extracted from tapes and imported into Sony Soundforge.

### Visual Display Characterization

Videos were assessed (resolution: 30 frames/sec), in order to record distinct behaviors. Information was compiled and displays of males (N = 11) from tethered female trails were used to construct ethograms. Courtship recordings with live females could not be easily analyzed, thus they were omitted for measurement purposes. In a subset of data where we could analyze displays, we observed no differences in courtship (data not shown). Measurements of rates and durations were averaged for each individual. An Iconico screen protractor (v3.3) was used on individual video frames to find angle ranges of the 3^rd^ legs of males (N = 5).

In addition, we also recorded displays of males (N = 3) with a high-speed camera (Photron fastcam SA3, 1000 frames/second) so that certain features of male movement could be more easily clarified. Using the larger rotating platform, we positioned the regular speed camera to a “female's eye view” and the high-speed camera with a side view of the courting male.

### Vibrational Signal Analysis

Laser recordings from each individual were filtered below 80 Hz in Sony Soundforge to remove background noise. For quantification of vibratory signal elements, 5 samples of each element were randomly selected across an individual's display and averaged. Duration, dominant (peak) frequency, and bandwidth (10 dB below peak frequency) were measured using custom written Matlab scripts (Mathworks Inc., v7.0.).

Only complete displays that progressed to a copulation attempt were used. In total, vibrations from 5 different males were scored. Coefficients of variation were calculated to quantify variation of signal elements within individuals and across the entire group sampled.

## Results

Total courtship time of *Maratus volans* ranged from 6–51 minutes (mean = 24.35±19.49 min, N = 5). Use of visual and vibrational signals varied although overall patterns in the sequence of mating behavior included many distinct, stereotyped behavioral elements that could be consistently identified across individuals.

### Visual Displays

For an initial review of *M. volans* displays and a comprehensive collection of images, refer to Hill [33]. Table 1 gives detailed descriptions, including contextual information, of distinct behaviors, and footage of most of these behaviors is provided in Video S1. Behaviors are presented in the general order of their appearance within a courtship sequence.

**Table 1:** Visual ethogram for *Maratus volans*.

Behavior	Description	Occurrence	Measurement
**pedipalp flicker** *	The pedipalps are brought together in the front of the carapace and moved up and down in unison.	intermittently throughout courtship	**Rate:** 3.5±1.6 flicks/sec (N = 5)
**opisthosomal bobbing** *	The abdomen, or opisthosoma, is moved up and down in a rapid manner. It can be parallel with the substrate or more vertically oriented, with fan flaps either splayed out (expanded) or folded around opisthosoma (retracted). During production, the male is stationary, with either all leg pairs contacting the substrate, or with the 3^rd^ legs in an erect leg wave stance.	(1) when males approached females from a distance, preceding all other courtship behaviors (excluding pedipalp flickering), (2) between bouts of leg waving/fan dancing.	**Number of bouts:** 2–11 (N = 5)**Bout duration:** 75.5±58.6 secs
**3^rd^ leg wave**	The 3^rd^ legs are swiftly extended and raised to an approximately vertical, erect leg wave stance. Almost immediately, the 3^rd^ legs, while still extended, are lowered and simultaneously brought back towards the abdomen slightly to form a bilateral “V”. At their lowest point, the 3^rd^ legs are flexed at the patella briefly before they are quickly rotated up and forward to their original position. This motion appears seamless and is repeated several times with no gaps between leg waves.	following several bouts of opisthosomal bobbing, while males are facing a female	**Bout duration**: 6.7±3.2 secs (N = 5)**Rate:** 3.2±0.6 waves/sec (N = 5)**Angle ranges:** (Fig. 2, N = 5)#1 = 36–71°#2 = 72–120°#3 = 124–168°
**fan dance** *	Open opisthosomal fan (flaps expanded) moves back and forth laterally, similar to a metronome, at varying speeds. Most often produced in synchrony with leg waving, but sometimes the male is stationary either with the 3^rd^ legs in an erect leg wave stance, or in contact with the substrate.	when male is in close proximity to a female	**Rate:** 3.2±1.5 cycles/sec**Angle range:** (Fig. 2, N = 5)#4 = 19–35°
**fan-flapping**	While opisthosoma is vertically oriented, fan flaps are extended and retracted several times in a sequence. The male is stationary with 3^rd^ legs in an erect leg wave stance.	periodically during pauses in movement that follow fan dances	**Rate:** 2.5±1.8 flaps/sec
**pre-mount display**	The 3^rd^ legs are rotated forward, and the carapace is brought up over the 1^st^ and 2^nd^ legs. Simultaneously, opisthosomal fan flaps are retracted and the abdomen is tilted until the posterior portion is close to the substrate. Regularly spaced bouts of opisthosomal bobbing follow, each of which correspond with tremors and lowering of 3^rd^ legs. Later, the 1^st^ legs are flexed and raised slightly while the 3^rd^ are rotated all the way down and behind the 1^st^ and 2^nd^ legs where they are held extended in an upside down “V”. The 1^st^ legs are now held erect out in front of the carapace and moved down closer to female during bouts of opisthosomal bobbing.	occurs at the end of the courtship display, immediately preceding and leading into a mounting attempt	**Duration:** 54.1±7.4 secs**Angle range:** (Fig. 4, N = 5)#5 = 126–148°

*after behavioral descriptions similar to that of two related species, *M. pavonis* and *M. splendens* [36].

#### Pedipalp flicker

Pedipalp flickers were observed to occur intermittently throughout the entire duration of courtship, alone or in conjunction with all other displays. During interactions between live individuals of both sexes, males performed pedipalp flickers even when females were not oriented in their direction; females were also observed doing this behavior. This behavior was common and occurred in other contexts (i.e. when individuals were feeding, or just moving about their individual containers alone), suggesting that pedipalp flickering may not be specific to the mating display. Regardless, it is included here as it is a prominent behavior during courtship. Pedipalp flickering ramps up in intensity immediately preceding movements, such as opisthosomal bobbing or leg waving.

#### Opisthosomal bobbing

Male peacock spiders can move their abdomen up and down independently of expanding and retracting opisthosomal flaps, and vise-versa [33]. Vibrational signals are associated with this movement (discussed at length in the “Vibrational Signals” section). High-speed video analysis revealed that in between “bobs”, lateral movements of the abdomen sometimes occur, particularly during the pre-mount display.

#### 3^rd^ leg wave

**Figure 2:**
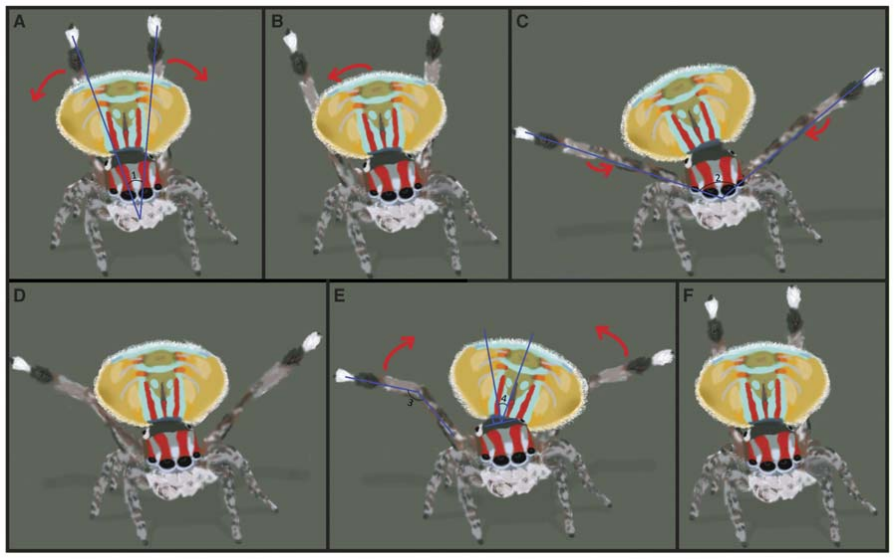
Fan dance of Maratus volans. (A) Males begin this display by swiftly raising the 3^rd^ legs to an erect leg wave stance. (B) Immediately after, extended 3^rd^ legs are lowered and (C) brought forward slightly until they are just above the top of the carapace. (D) At this point 3^rd^ legs are slightly bent at the patella and (E) quickly raised until they are returned to their initial position. One cycle of fan dancing occurs between (B) and (F). Angle measurements (#1-4) are provided in Table 1.

The majority of a male peacock spider's courtship display is comprised of 3^rd^ leg waves (Fig. 2a–f). Similar to jumping spiders in the *Habronattus* genus, the 3^rd^ legs of *Maratus* males are elongated and ornamented relative to the other pairs [33]. Specifically, the metatarsi of *M. volans'* 3^rd^ legs are covered with a dense tuft of black setae and a comparably thick clump of white setae adorn the tarsi [33]. If not already in position, leg waving begins with the male raising his 3^rd^ legs into the air rapidly. Sometimes simultaneously, or shortly after, the opisothoma is lifted and flaps are unfurled, though often, especially if the male is further from the female, leg waves precede this expansion of the “fan.” Once the 3^rd^ legs are brought upright, they are immediately lowered, while remaining extended (Fig. 2a–e). They are only flexed at the patella when they crest the bottom of their rotation and are smoothly re-extended as they return to a vertical position. Rotation of the 3^rd^ legs is most likely around the coxa-trochanter joint [35], although this could not be confirmed.

Leg waving usually occurs bilaterally, but occasionally one 3^rd^ leg was waved completely on its own (this was observed for both right and left legs), or each side was waved asynchronously, in an alternating fashion. Bouts of leg waving occur intermittently, sometimes while a male is stationary, but often, 3^rd^ leg waving occurs in conjunction with a side-stepping motion akin to the side-to-side motion that occurs during the fan dance of *Maratus pavonis* and *Maratus splendens* [36]. High-speed video analysis revealed that the male takes each side step as the 3^rd^ legs pass through the lowest part of a leg wave. A male will move in semi circles around the female, going in one direction for a while before heading back the way he just came, getting slightly closer to where she is standing with each shift in direction. During this side stepping, the 3^rd^ leg in the direction of movement is commonly held perpendicular to the substrate while the other leg was held slightly lower (albeit still extended) and waved much more intensely. Hill and Otto [36] also observed this tendency in *Maratus pavonis*.

#### Fan dance

The opisthosomal fan is the feature for which spiders in the *Maratus* genus are named [37]; accordingly, the fan dance is the most notable aspect of this spider's courtship. High-speed video analysis revealed that when a male is fan dancing in conjunction with leg waving (Fig. 2c–e), each cycle of fan movement reliably corresponds with a single 3^rd^ leg wave (Fig. 2a–f). The closer a male is to a female, the more likely he is to adopt a “hunker-down” pose when performing a stationary fan dance. This stance is characterized by lowering of the carapace, almost to the ground, and bending of the front legs more sharply at the patella to bring them tight against the carapace; the 3^rd^ legs remain in an erect in “V” position. While still in this pose, males will regularly follow fan dancing with some opisthosomal bobbing.

#### Fan flapping

**Figure 3:**
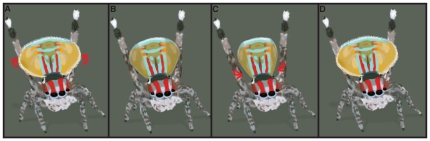
Fan-flapping of *M. volans*. (A) 3^rd^ legs in erect leg wave stance, (B) initial retraction of the distal portion of the flaps commences. (C) Flaps are further contracted before (D) being quickly expanded again.

Males often pause after a bout of 3^rd^ leg waving/fan dancing, seemingly to gauge female attention and/or intention. Sometimes during these pauses, especially if a female is not oriented directly in front of males any more (either by his or her movement), males will slowly flutter the portion of the opisthosomal fan that can be tucked around the abdomen for a few seconds (Fig. 3a–d). In the context that it was seen to occur, fan flapping is potentially a means of drawing attention back to the male. Indeed in several cases, fan flapping elicited such a response by females who would reorient themselves towards the male after he performed this behavior. During courtship displays evoked using pinned dead females, a male would flap his fans until the female was swiveled slightly, in a manner to mimic a female tracking male movement, at which point the male would commence fan dancing again.

#### Pre-mount display

**Figure 4:**
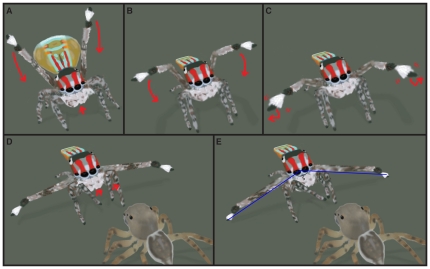
Pre-mount display of *M. volans*. (A) From an initial leg wave stance, the 3^rd^ legs are rotated forward and (B), (C) the carapace is brought up over the 1^st^ and 2^nd^ legs. Simultaneously, opisthosomal fan flaps are retracted and the abdomen is tilted until the posterior portion is close to the substrate. (D) Regularly spaced tremors following paired lowering of the 3^rd^ legs. Once legs are lowered to be approximately parallel to the substrate, the 1^st^ legs are flexed and raised slightly while (E) the 3^rd^ legs are rotated all the way down and held extended in an upside down “V” behind the 2^nd^ legs. The 1^st^ legs are now held erect out in front of the carapace and gradually moved down closer to female. Angle measurement (#5) is provided in Table 1.

In contrast with the majority of the male peacock spider's courtship display, the pre-mount display proceeds in a precisely stereotyped sequence (Fig. 4). To begin, the 3^rd^ legs are rotated to the front of the carapace, which is brought forward and uplifted over the 1^st^ and 2^nd^ legs (Fig. 4a–b) at the same time. Simultaneously, the opisthosomal fan flaps are retracted and the abdomen is tilted until the anterior portion is level with the top of the carapace and the posterior portion is fairly close to the substrate. Regularly spaced bouts of opisthosomal bobbing follow, each of which correspond with tremors of extended 3^rd^ legs (Fig. 4c); this aspect of the display is discussed more thoroughly under the “Vibrational Signals” section below. During tremors, the 3^rd^ legs are moved up and down only slightly, but very rapidly. At the end of a tremor, the 3^rd^ legs are lowered and spread further apart than when they started. When the 3^rd^ legs are lowered to about the top of the carapace, after approximately 3–8 tremors, the 1^st^ legs are flexed and raised slightly off the substrate (Fig. 4d). During the next tremor, the 3^rd^ legs are rotated all the way down in front of the carapace and continue to be rotated back behind the 1^st^ and 2^nd^ legs where they are held extended in an upside down “V” (Fig. 4e). The 1^st^ legs are then held erect out in front of the body at carapace level almost touching the female, termed the “glider pose”. Now with each bout of opisthosomal bobbing the male bends the tarsi and metatarsi of the 3^rd^ legs slightly, and moves the 1^st^ legs down closer to the female until finally touching her carapace. It was previously inferred [33] that the 1^st^ legs do not play a role in the visual courtship display of these spiders, but this is not true of the pre-mount display. The time between opisthosomal bobbing bouts continues to decrease at this point and in time, the male makes advances over the top of the female's carapace towards her abdomen.

### Vibrational Signals

In general, vibrations are extensively utilized throughout male courtship and are often a precursor to motion displays, especially when the female was at a distance and/or not oriented directly at a male. Vibrations are caused by oscillation of the abdomen [15], [38], and indeed, analysis of video recordings demonstrated that all vibrational signals coincide with opisthosomal bobbing; vibrations were absent during any lateral movement of the abdomen between bouts of bobbing. It remains unclear which mechanisms males are using to produce vibrations though. Stridulation of paired structures on their abdomen and cephalothorax may be employed. Vibrations might also be generated by tremulation, that is, abdominal oscillations transferred directly to the substrate via the animals' legs [15]. Further study using synchronous high-speed video/laser vibrometry and ablation experiments are needed.

Vibrational signals can be broken down into two categories: (1) those that occur immediately and continue intermittently throughout the majority of the display, termed **rumble-rumps**, and (2) those that occur during the pre-mount display only, which include (a) **crunch-rolls**, and (b) **grind-revs**. An example of each signal is audible, albeit softly, in Video S1.

Some vibrational signals are comprised of several components. Not all vibrational signals correspond with visual displays. For ease of discussion, signaling elements have been given names according to the acoustic characteristics of that signal. Table S1 summarizes properties of each signal element and for each measure, quantifies variation seen within and between individuals. As a general trend, variation in signal elements was greater for the group than within an individual for dominant frequency. The opposite trend was observed for signal duration.

#### 1. Rumble-rumps (Rb-Ru)

Rumble-rumps are the most common signals produced during courtship, seemingly as soon as a male detects the presences of a female, and even at long-distances. Rumble-rumps are short in duration (2.44±0.28 sec, range 2.20–2.80 sec, N = 5). Intervals between signals are usually longer than the signals themselves (3.46±2.48 sec, range 0.19–7.17 sec, N = 5). The mean number of *Rb-Ru's* in a bout is 17.4±12.8 (range = 4–39, N = 5). *Rb-Ru* bout numbers and duration correspond with that of opisthosomal bobbing, as reported in Table 1.

**Figure 5:**
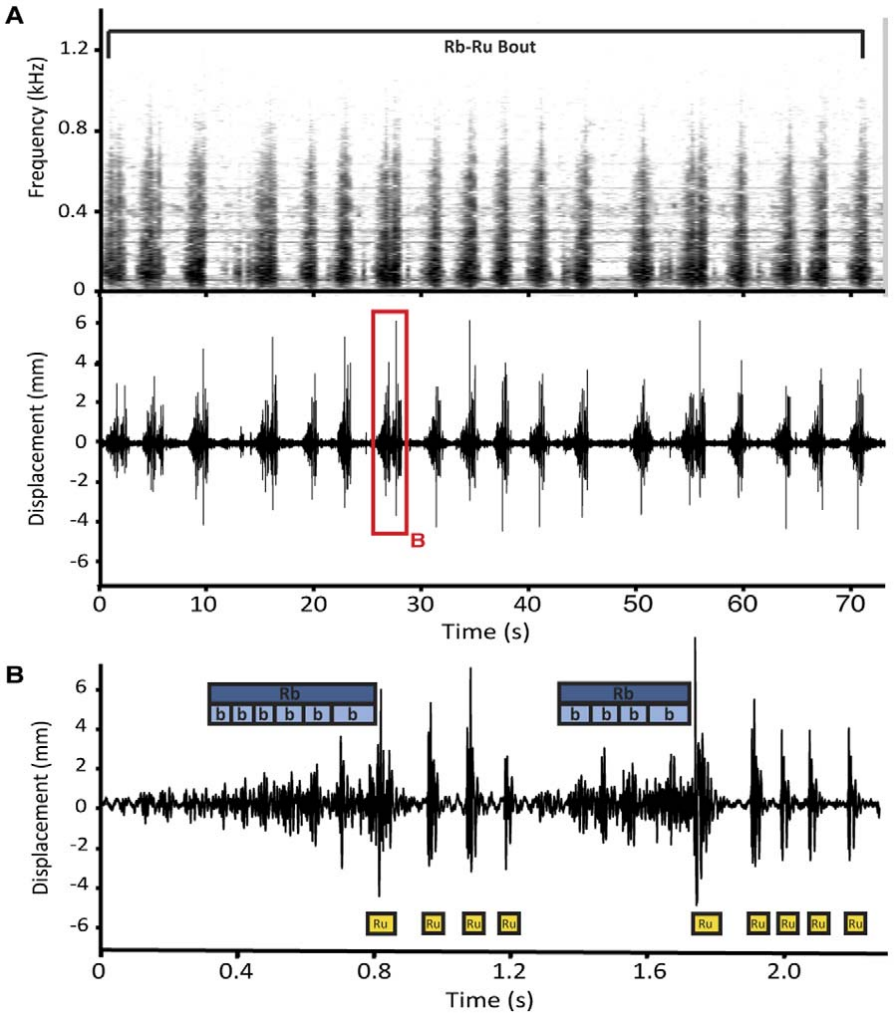
Substrate borne signals of courting male *M. volans*. (A) Spectrogram (window size = 26422) and waveform of a bout of rumble-rumps. (B) Waveform of a single rumble-rump. Substrate-borne signals occur throughout the M. volans display.

Rumble-rumps are composed of two distinct elements (Fig. 5b, Table S1), “rumbles” (*Rb*) and “rumps” (*Ru*), although, there is considerable variation in the way that rumble rumps are put together. All *Rb-Ru's* start with a *Rb*, followed by 1–5 (typically 3) *Ru's*. This is annotated as follows: *Rb Ru^1–5^*. Other rumble-rump combinations observed include: *Rb Ru^1–5^ Rb*, and *Rb Ru^1–5^ Rb Ru^1–5^*. An example of a longer *Rb-Ru* (*Rb Ru^4^ Rb Ru^5^*) is shown in Fig. 5b.

Each *Rb* is made up of 3–8 “bumps” (*b*) (Fig. 5b, Table S1), which occur at a mean rate of 12.1±2.9 bumps per second (N = 5). The interval between *Ru's* in a rumble-rump ranges from 0.07–0.62 seconds (mean = 0.25±0.26 sec, N = 5). As a general rule, *Ru's* that occur immediately at the end of a *Rb* are usually highest in amplitude. *Ru-Rb's* continue to be produced during breaks in fan-dancing and leg waving until the pre-mount display begins.

#### 2a. Crunch-rolls (Cr-Roll)

**Figure 6:**
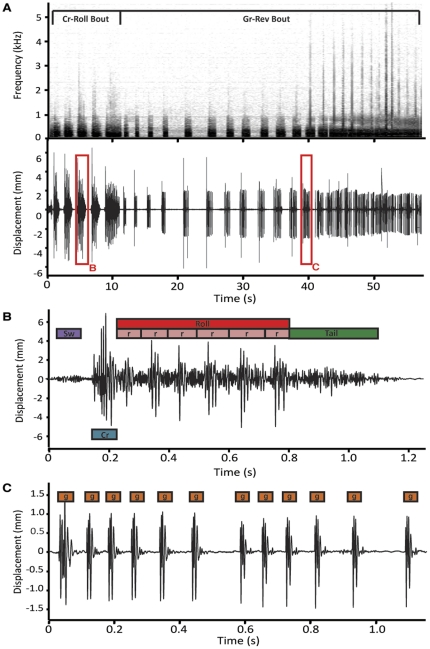
Pre-mount display substrate-borne signals of courting male *M. volans*. (A) Spectrogram (window size = 11206) and waveform of a bout of crunch-rolls and grind-revs as they occur in sequence. (B) Waveform of a single crunch-roll. (C) Waveform of a single grind-rev. Crunch rolls and grind revs are produced exclusively during pre-mount displays.

The first *Cr-Roll* signal is always preceded by a very brief (mean = 0.21±0.19 sec) intro signal, which is just visible in the waveform in Fig. 6a. It was difficult to determine if this intro was a signal or a byproduct of adopting the pre-mount display position (Fig. 4a–b), when a male's legs and body are simultaneously raised. Either way, it was the lowest frequency vibration observed, with a peak frequency of 84±18 Hz (N = 5).

*Cr-Rolls* are the vibrational signals produced during the opisthosomal bobbing and leg tremors that occur at the beginning of the pre-mount display. *Cr-Roll* signals consist of “swish” (*Sw*), “crunch” (*Cr*), “roll” (*Roll*), and “tail” (*Tail*) elements (Fig. 6b, Table S1). *Sw's* occur at the beginning of *Cr-Roll's* and are quickly followed by a single *Cr.* Some *Cr-Roll's* don't have *Sw's*, typically the first and last *Cr-Roll's* of a bout, but in *Cr-Roll's* that do (74.6±7.7% of Cr-Rolls), high-speed video revealed that the tarsi of 3rd legs are flicked down at the end of each Sw and brought back up at the beginning of each *Cr. Roll's* follow *Cr's* and are paired with the swift and shallow flick of 3rd legs at the patella. During the second or third to last *Cr-Roll*, a male lifts legs I off the substrate (Fig. 4d). During the last *Roll* of the last *Cr-Roll*, a male would lower 3rd legs to a near upside-down “V” position, while the body was again lifted and legs I were further outstretched in front of the carapace. *Roll's* in all but the last *Cr-Roll* are made up of 3–7 (mean = 5.5±2.1, N = 5) smaller elements (r's) (Fig. 6b, Table S1), which occur at a rate of 9.6±0.3 *r's* per second (N = 5). *Roll's* in the last *Cr-Roll* were much longer and included a mean of 17.9±2.1 *r's*. Also, in contrast with the other *Cr-Roll's*, the last *Cr-Roll* signal in a bout never had a *Tail* portion.

On average, a single *Cr-Roll* was 1.32±0.13 seconds (N = 5), and the interval between *Cr-Roll's* was 0.88±0.37 seconds. Bouts of *Cr-Roll's* lasted a mean of 14.13±5.00 (N = 5) seconds and included 5–11 (mean = 7.2+1.8, N = 5) individual *Cr-Roll's*. The mean interval between the end of the last *Cr-Roll* and the first grind-rev signal was 0.71±0.26 seconds (N = 5).

#### 2b. Grind-revs (Gr-rev)

Grind-revs are produced in the final stages of the pre-mount display and continue to occur as a male mounts and attempts to copulate with a female. As seen with *Cr-Rolls*, leg movement is highly coordinated with *Gr-rev* vibrations. When a male begins *Gr-rev* signal production, he is in the “glider pose” (Fig. 4e), that is, 3rd legs are in a downward “V” position and the 1^st^ legs are extended out in front of the carapace. With each successive *Gr-rev* signal, legs I are brought closer together at the most distal end and lowered (albeit still extended) nearer to the female's carapace. At this point, she is now positioned almost directly below the male's leg I tarsi. Once he is touching the female, the male begins to move over the female's carapace towards her abdomen in time with *Gr-rev's*.

An average bout of *Gr-rev's* lasts for 38.77±19.08 seconds (N = 5) and includes anywhere from 12–34 *Gr-rev's* (mean = 26.9±6.4, N = 5). However, it is difficult to distinguish individual *Gr-rev's* approaching the finale of the pre-mount display as the interval between *Gr-rev's* becomes increasingly small (mean = 1.04±0.30 sec, range = 0.67–1.34 sec, N = 5). As a general rule, during the progression of the pre-mount display the duration of individual *Gr-rev's* (range = 0.16–0.81 sec, N = 5) and the interval between each increases and decreases, respectively, as a male advances towards a female. The spectrogram of *Gr-rev's* included much higher frequencies than all other signals measured, (Fig. 5a).

*Gr-rev's* are composed of a sequence of “grinds” (*g*) (Fig. 6c, Table S1), which are emitted in groups of 3–11 (mean = 8.54±0.23, N = 5) at a rate of 12.64±3.88 *g's* per second, N = 5); again, these groups blur at the end of the pre-mount display as the interval between *Gr-rev's* becomes increasingly small. In contrast, throughout a bout of *Gr-rev's*, intervals between *g's* remain fixed around a mean of 0.05±0.10 seconds (N = 5). At the end of a *Gr-rev* bout, males attempted to copulate with the female (or female model).

## Discussion

Our results show that peacock spiders use visual displays in conjunction with vibratory signals during courtship. The full repertoire of these males is truly remarkable, particularly the visual components. While visual and vibrational signals are variable between males, some overall patterns were evident. For instance, male courtship usually began with rumble-rump vibrations produced at a distance. When males get close to females, they begin to perform multimodal displays, primarily 3^rd^ leg waving and fan dancing (visual) along with rumble-rumps (vibrational). Males add new elements as courtship progresses to the finale, specifically the pre-mount display and associated crunch-roll and grind-rev vibratory signals. Unfortunately, our study was limited by the number of males and complete displays we were able to capture. Larger samples are needed to assess more accurately the amount of natural variation in courtship that exists for individual males, as well as within and between wild populations of these spiders.

It should be noted that even with complete video footage of male courtship, precisely characterizing displays is still a challenge. This is especially the case when trying to pick out features that might be important to animals with sensory systems unlike our own. Display and signal elements were quantified as thoroughly as possible, but the former should only be treated as estimates. The sequence of animal behaviors is often influenced by many dynamic factors in the wild, and at this point it is difficult to accurately predict if the sequence and durations of individual behaviors observed in the lab are as similar to that which would be seen in nature. Our immobile female models offered us the freedom to easily document male displays, however, the progression of courtship is unlikely to progress in such a simple manner. In the case of these spiders, female feedback, in the form of receptivity and/or aggression (often seen in trials between both live sexes) undoubtedly contributes greatly to the way in which males proceed in their courtship efforts.

While both visual and vibratory signals are well demonstrated as being important in mating systems of spiders [9], [15], our understanding of complex multimodal signals is still in the very early stages [39]. The adaptive significance of multi-modal signal structure remains poorly studied [39], as are mechanisms by which sexual selection operates on multi-modal signals [2]. Natural signaling habitats are rarely homogeneous and therefore provide a variety of signaling channels and strategies to exploit, each of which may vary contextually in their efficacy of information transfer. Not surprisingly, evidence suggests that in a mating context, males may actually use multiple different signal strategies, alone or in conjunction, in response to varying abiotic and biotic factors [1], [40]–[42].

We observed that *Maratus volans* males use vibratory signals over visual displays at long distances. In contrast, one well studied example from the North American genus *Habronattus*, *H. dossenus* only uses vibrational signals at close proximities to females, mainly using visual displays when greater than 5–8 mm away from females. Presumably *H. dossenus* behavior is adapted to variable vibratory environments [24]. Our descriptions might then suggest that the visual environment of *Maratus* is more heterogeneous than the signaling substrate, and thus offers a better channel for communication over a greater distance. Future work on the ecology, habitat usage patterns, and signaling environment of *Maratus* species will test this hypothesis. Given that multimodal signal structure is a most likely a product of a combination of dynamic selection pressures [1], [43], some of the plasticity observed in *M. volans* displays could be favored by selection in order to minimize costs of signals production and maximize success in variable environments.

Although *Maratus* has evolved independently from *Habronattus*, the two genera possess several similarities in morphology, behavior, and habitat. Specifically, species of *Maratus* and *Habronattus* share elongated 3^rd^ legs, have similarly structured genitalia, and are primarily ground-dwelling [33]. Both genera also show a remarkable amount of interspecific morphological diversity [6], [26], [30], [33]–[34], [36] and much diversity remains to be discovered in *Maratus* and its relatives. Direct comparisons of complex signals in these two genera will better inform our understanding of multi-modal signal structure and function. For instance, while *Maratus* and *Habronattus* both make use of multimodal signals, *Maratus* vibrational signals are relatively simple compared to those seen in some *Habronattus* species [17], [26]–[27]. Instead, *Maratus volans* males invest more in their ornamentation and visual displays (as evidenced by the evolution of the opisthosomal flaps). This pattern is predicted in several models of multiple signal evolution and has been empirically shown in several groups of birds [44]–[46]. The opposite pattern from *Maratus volans*, where vibrations are emphasized over visual displays, is seen in some closely related species from the genus *Lycidas* [47]. In work on *Lycidas michaelseni* (*Saitus michaelseni* in [18]), behavioral observations suggested that males primarly courted females using audible substrate-borne signals. Interestingly, visual signals were deemphasized as males stridulated directly above female nests out of view [18]. This type of behavior also occurs in several other *Lycidas* species found in the same habitats as *M. volans* (Girard and Elias, unpublished observations). It is quite possible that signal complexity may be limited by evolutionary tradeoffs where investment in one modality necessitates reduction in another [48]–[51] and this might explain the pattern observed for greater visual complexity in *Maratus* multi-modal signals.

Despite their common occurrence in nature, multi-modal signals have received relatively little attention thus far (but see [1], [6], [39]). Female choice has long been the subject of in-depth investigations, but we are only now starting to use this framework to examine complex multimodal signals. Jumping spider communication offers an excellent system to study behavior and the role of sexual selection in the evolution of species and mating systems. Specifically, the *Maratus* genus lends itself as a perfect system for such studies. In conclusion, this study provides the foundation necessary for future research on the *Maratus* genus and the evolution of complex signals.

## Supporting Information

Video S1 **Example display elements from** ***M. volans*** **male courtship behavior.** In order of appearance: fan raise with expansion of fan flaps; opisthosomal bobbing and concurrent rumble-rump vibrations; simultaneous fan dance and 3^rd^ leg wave; pre-mount display with associated crunch-roll and grind-rev vibrations.
(M4V)

Table S1 Mean measurements of distinct vibrational signal elements of *M. volans* males (N = 5), which are color coded to correspond with Figures 4 and 5. Coefficients of variation (CV) were calculated to quantify variation as it was observed within individuals (Ind.) and across the entire group sampled (Group). Frequency characteristics of *Rb's* and *Roll's* are similar to that of their components, *b's* and *r's*, respectively.
(TIF)

## References

[1] Hebets E, Papaj D (2005). Complex signal function: developing a framework of testable hypotheses.. Behavioral Ecology and Sociobiology.

[2] Candolin U (2003). The use of multiple cues in mate choice.. Biological reviews of the Cambridge Philosophical Society.

[3] Rowe C (1999). Receiver psychology and the evolution of multicomponent signals.. Animal Behaviour.

[4] Huber B (2005). Sexual selection research on spiders: progress and biases.. Biological Reviews.

[5] Johansson B, Jones T (2007). The role of chemical communication in mate choice.. Biological Reviews.

[6] Elias D, Hebets E, Hoy R, Mason A (2005). Seismic signals are crucial for male mating success in a visual specialist jumping spider (Araneae: Salticidae).. Animal Behaviour.

[7] Scheffer S, Uetz G, Stratton G (1996). Sexual Selection, Male Morphology, and the Efficacy of Courtship Signalling in Two Wolf Spiders (Araneae: Lycosidae).. Behavioral Ecology and Sociobiology.

[8] Taylor L, McGraw K (2007). Animal coloration: sexy spider scales.. Current Biology.

[9] Foelix R (1996). Biology of Spiders, 2nd edn.

[10] Clark D, Morjan C (2001). Attracting female attention: the evolution of dimorphic courtship displays in the jumping spider *Maevia inclemens* (Araneae: Salticidae).. Proc R Soc Lond Ser B-Biol Sci.

[11] Hill D, Richman D (2009). The evolution of jumping spiders (Araneae: Salticidae): a review.. Peckhamia.

[12] Li J, Zhang Z, Liu F, Liu Q, Gan W (2008). UVB-based mate-choice cues used by females of the jumping spider *Phintella vittata*.. Curr Biol.

[13] Lim M, Li J, Li D (2008). Effect of UV-reflecting markings on female mate-choice decisions in Cosmophasis umbratica, a jumping spider from Singapore.. Behav Ecol.

[14] Maddison W, Hedin M (2003). Jumping spider phylogeny (Araneae: Salticidae).. Invertebrate Systematics.

[15] Uhl G, Elias D, Herberstein M (2011). Communication.. Spider behavior: flexibility and versatility.

[16] Edwards G (1981). Sound production by courting males of *Phidippus mystaceus* (Araneae: Salticidae).. Psyche.

[17] Elias D, Mason A, Maddison W, Hoy R (2003). Seismic signals in a courting male jumping spider (Araneae: Salticidae).. Journal of Experimental Biology.

[18] Gwynne D, Dadour I (1985). A new mechanism of sound production by courting male jumping spiders (Araneae: Salticidae, *Saitis michaelseni* Simon).. Zoological Society of London.

[19] Jackson R, Witt P, Rovner J (1982). The behavior of communicating in jumping spiders (Salticidae).. Spider Communication: Mechanisms and Ecological Significance.

[20] Maddison W, Stratton G (1988). Sound production and associated morphology in male jumping spiders of the *Habronattus agilis* species group (Araneae, Salticidae).. Journal of Arachnology.

[21] Maddison W, Stratton G (1988). A common method of sound production by courting male jumping spiders (Araneae, Salticidae).. Journal of Arachnology.

[22] Noordam A (2002). Abdominal percussion and ventral scutum in male *Euophrys frontalis* (Araneae: Salticidae).. Entomologische Berichten, Amsterdam.

[23] Sivalinghem S, Kasumovic M, Mason A, Andrade M, Elias D (2010). Vibratory communication in the jumping spider Phidippus clarus: polyandry, male courtship signals, and mating success.. Behav Ecol.

[24] Elias D, Mason A, Hoy R (2004). The effect of substrate on the efficacy of seismic courtship signal transmission in the jumping spider *Habronattus dossenus* (Araneae: Salticidae).. Journal of Experimental Biology.

[25] Elias D, Hebets E, Hoy R (2006). Female preference for signal complexity/novelty in a jumping spider.. Behavioral Ecology.

[26] Elias D, Hebets E, Hoy R, Maddison W, Mason A (2006). Regional seismic song differences in sky-island populations of the jumping spider *Habronattus pugillis* Griswold.. J Arachnology.

[27] Elias D, Land B, Mason A, Hoy R (2006). Measuring and quantifying dynamic visual signals in jumping spiders.. Journal of Comparative Physiology A Sensory Neural and Behavioral Physiology.

[28] Andersson M (1994). Sexual Selection.

[29] Maddison W, Hedin M (2003). Phylogeny of Habronattus jumping spiders (Araneae: Salticidae), with consideration of genital and courtship evolution.. Systematic Entomology.

[30] Maddison W, McMahon M (2000). Divergence and reticulation among montane populations of a jumping spider (*Habronattus pugillis* Griswold).. Systematic Biology.

[31] Masta S (2000). Phylogeography of the jumping spider *Habronattus pugillis* (Araneae: Salticidae): recent vicariance of sky island populations?. Evolution.

[32] Masta S, Maddison W (2002). Sexual selection driving diversification in jumping spiders.. PNAS.

[33] Hill D (2009). Euophryine jumping spiders that extend their third legs during courtship (Araneae: Salticidae: Euophryinae: *Maratus*, *Saitis*).. Peckhamia.

[34] Otto J, Hill D (2010). Observations of courtship display by a male *Maratus amabilis* Karsch 1878 (Araneae: Salticidae).. Peckhamia.

[35] Parry D (1957). Spider leg muscles and the autotomy mechanism.. Scientifc Monthly.

[36] Hill D, Otto J (2011). Visual display by male *Maratus pavonis* (Dunn 1947) and *Maratus splendens* (Rainbow 1896) (Araneae: Salticidae: Euophryinae).. Peckhamia.

[37] Dunn R (1947). A new salticid spider from Victoria.. Memoires of the National Museum of Victoria.

[38] Elias D, Mason A, O'Connel-Rodwell C (2011). Signaling in variable environments: Substrate-borne signaling mechanisms and communication behavior in spiders.. The Use of Vibrations in Communication: properties, mechanisms and function across taxa.

[39] Partan S, Marler P (2005). Issues in the classification of multimodal communication signals.. American Naturalist.

[40] Coleman S, Patricelli G, Borgia G (2004). Variable female preferences drive complex male displays.. Nature.

[41] Endler J (1992). Signals, signal conditions, and the direction of evolution.. American Naturalist.

[42] Endler J (1990). On the measurement and classification of color in studies of animal colour patterns.. Biological Journal of the Linnean Society.

[43] Bro-Jørgensen J (2010). Dynamics of multiple signalling systems: animal communication in a world in flux.. Trends in Ecology & Evolution.

[44] Badyaev A, Hill G, Weckworth B (2002). Species divergence in sexually selected traits: Increase in song elaboration is related to decrease in plumage ornamentation in finches.. Evolution.

[45] Shutler D, Weatherhead P (1990). Targets of sexual selections - Song and plumage of wood warblers.. Evolution.

[46] Snell-Rood E, Badyaev A (2008). Ecological gradient of sexual selection: elevation and song elaboration in finches.. Oecologia.

[47] Zabka M (1987). Salticidae (Araneae) of Oriental, Australian and Pacific regions, II. Genera *Lycidas and Maratus*.. Ann Zool.

[48] Gibson J, Uetz G (2008). Seismic communication and mate choice in wolf spiders: components of male seismic signals and mating success.. Animal Behaviour.

[49] Iwasa Y, Pomiankowski A (1994). The evolution of mate preferences for multiple sexual ornaments.. Evolution.

[50] Johnstone R (1996). Multiple displays in animal communication: ‘Backup signals’ and multiple messages'.. Philosophical Transactions of the Royal Society of London Series Biological Sciences.

[51] Pomiankowski A, Iwasa Y (1998). Runaway ornament diversity caused by Fisherian sexual selection.. Proceedings of the National Academy of Sciences of the United States of America.

