# Supplementary figures and images for: Multi-Modal Courtship in the Peacock Spider, Maratus volans (O.P.-Cambridge, 1874)
Source: PLoS One. 2011 Sep 27;6(9):e25390. doi: 10.1371/journal.pone.0025390 (PMC3181266; doi:10.1371/journal.pone.0025390)

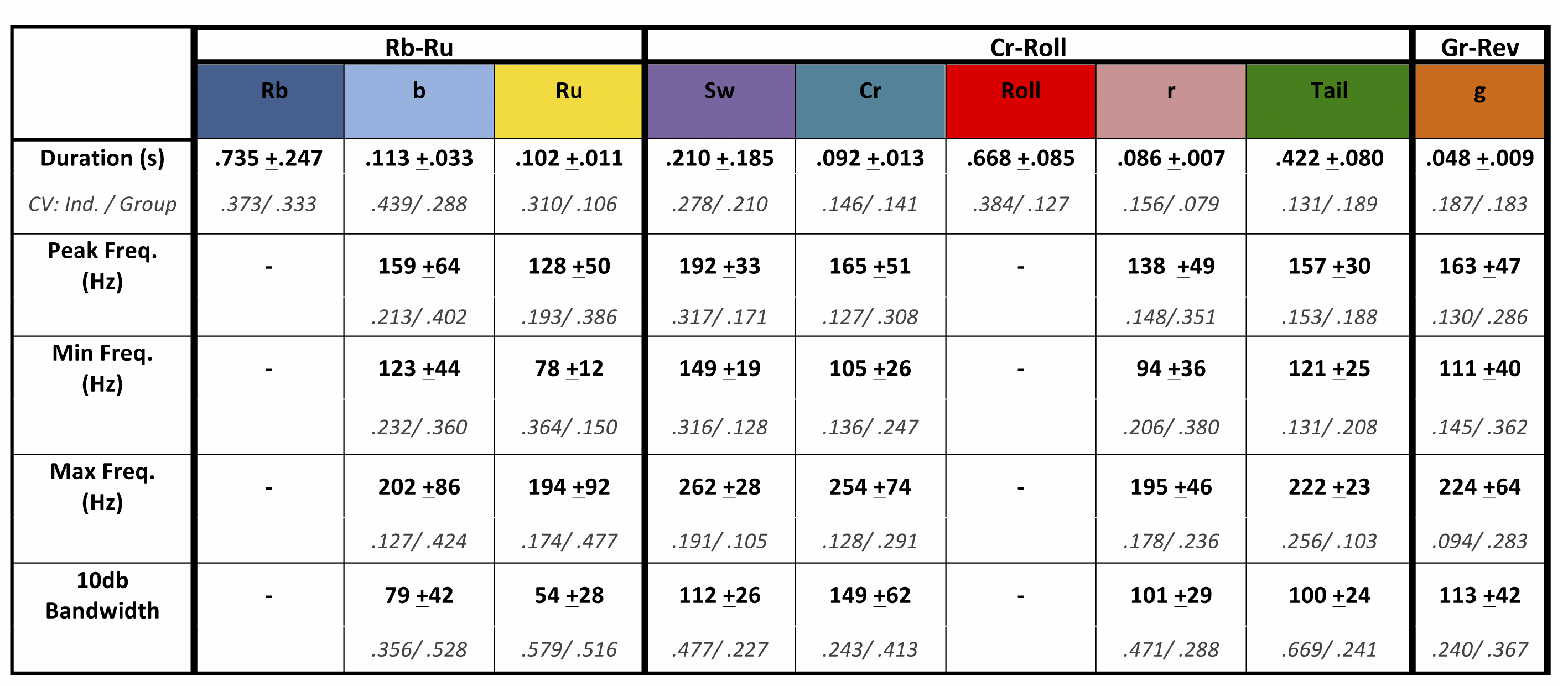

Supplement: Table S1 — Mean measurements of distinct vibrational signal elements of M. volans males (N = 5), which are color coded to correspond with Figures 4 and 5. Coefficients of variation (CV) were calculated to quantify variation as it was observed within individuals (Ind.) and across the entire group sampled (Group). Frequency characteristics of Rb's and Roll's are similar to that of their components, b's and r's, respectively. (TIF) [file pone.0025390.s002.tif]
